# Persisting Reversed Clock Syndrome

**DOI:** 10.1155/2005/463428

**Published:** 2006-02-27

**Authors:** Christophe Orssaud, Philippe Halimi, Claire Le Jeunne, Jean Louis Dufier

**Affiliations:** ^1^Consultation of OphthalmologyHôpital Européen Georges PompidouAssistance Publique – Hôpitaux de Paris20Rue Leblanc75015 ParisFrance; ^2^Department of RadiologyHôpital Européen Georges PompidouAssistance Publique – Hôpitaux de Paris20Rue Leblanc75015 ParisFrance; ^3^Department of Internal MedicineHôtel Dieu de ParisAssistance Publique – Hôpitaux de Paris1Place du Parvis Notre Dame75004 ParisFrance; ^4^Department of OphthalmologyHôpital Necker – Enfants MaladesAssistance Publique – Hôpitaux de Paris149Rue de Sèvres75015 ParisFrance

## Abstract

*Background:* The reversed clock phenomenon results in the transposition of objects from one side to another. Its major manifestation consists in the reversal of clock numbers in clock-drawing test. It could be due to a stroke disrupting attentional cerebral network. This phenomenon usually regresses in a few days.

*Objective:* To report a case of reversed clock phenomenon with disorders of space representation that did not regress spontaneously.

*Design:* Case report. Patient: A 67 year-old woman was referred due to headaches associated with gait disorder, visual field deficit and disturbance of space representation.

*Results:* Magnetic resonance imaging demonstrates two right cerebral infarcts mainly localized in the parieto-occipital region. A week after her stoke, clinical testing confirms a reversed clock phenomenon. The patient placed the hands of a clock in the opposite direction of what was specified. She got lost at home locating rooms in directions opposite to their real ones. Rehabilitation sessions partially improved these manifestations.

*Conclusion:* Although it usually improves in a few days, reversed clock phenomenon can persist longer. Rehabilitation sessions based on localization exercises may be helpful in such situations.

## 1. Introduction

The reversed clock syndrome (RCS) is an uncommon syndrome. Its most evident manifestation consists in a reversed placement of the numbers on a clock without omission during the clock-drawing task [1,2]. But, these reversals affect also the remembered space as revealed by mental imagery tasks. When patients are asked to describe a familiar place, they transpose rooms or objects from one side to the other. The patho-physiogenic mechanisms are not yet completely explained. Regression is spontaneous after few days according to the literature [1].

We would like to report the observation of a patient who presented a RCS due to a lesion of the right cerebral hemisphere associated with disorders of the space representation based on visual (or external) cues as well as on memorized (or internal) data. This RCS did not regress spontaneously, needing rehabilitation to improve. Mental imagery disorders were still present 18 months after the stroke.

### 1.1. Observation

Mrs B.P., 67 year-old woman, treated for arterial hypertension, obesity (BMI = 40%) and diabetes mel-

ISSN 0953-4180/05/$17.00 © 2005 – IOS Press and the authors. All rights reserved

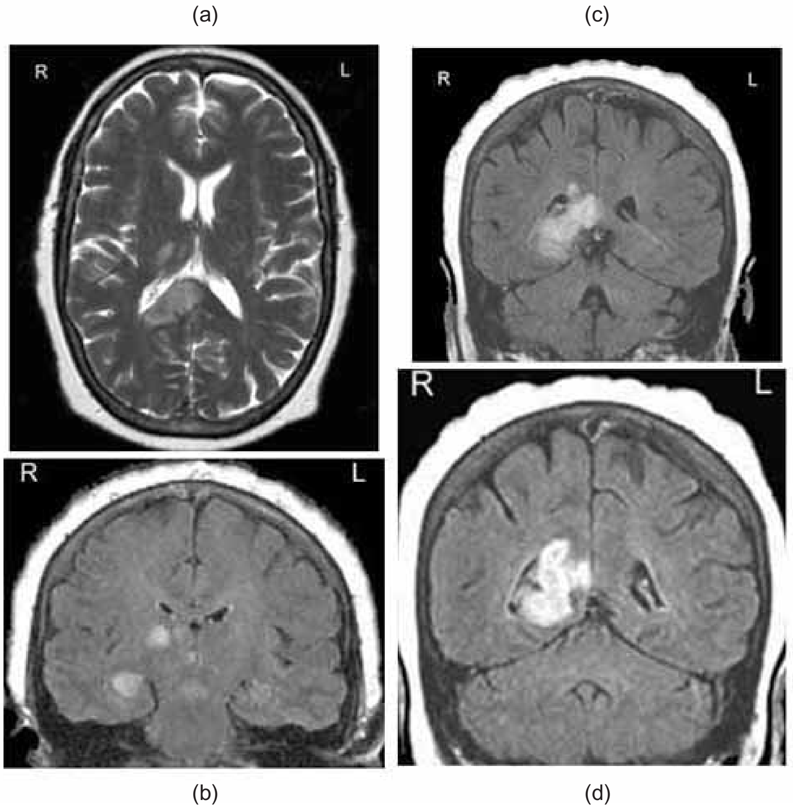

Fig. 1. n°1: MR performed 5 days after the stroke. a- Frontal T2 image shows 2 hyperintense lesions corresponding to infarcts involving right hippocampus and the right parieto-occipital region. b- Coronal T2 weighted flair image shows 2 hyperintense lesions corresponding to infarcts involving right hippocampus and thalamus. c- Coronal T2 weighted flair image shows hyperintense lesion corresponding to infarcts involving right parieto-occipital region. The lesion extent to the occipital part of the lateral ventricle. d- Coronal T2 weighted flair image on a more anterior surface than the Fig. 1(c).

litus, was refereed to the Hospital because of the occurrence of headaches associated with unsteady gait and falls. These neurological manifestations appeared a few hours beforehand. An enhanced brain CT scan was considered as normal. The patient was discharged.

Two days later, Mrs. P was admitted because of the persistence of the headache and the occurrence of a left homonymous heminanopsia. Neuro-radiologic examinations revealed two cerebral infarcts. The smallest one was localised in the territory of the right calcarine cortex. The second one was localised in the right anterior choroidal artery territories. This infarct involved the right internal parieto-occipital area, extending to the splenium of the corpus callosum, the adjacent white matter and the right hippocampal area (Fig. 1). The internal and posterior part of right thalamus was also included in this ischaemic process. The patient presented with gait disorder, a left homonymous hemianopsia and deficit of her internal and external space representation; otherwise, the neurological examination was normal. She explained that she always placed rooms in the opposite direction compared to their real ones. A week after the stroke, the patient was discharged from the hospital. The gait disorder had resolved. However, Goldman testing confirmed the persistence of the left homonymous hemianopsia and mental imagery was still abnormal. The patient was referred to the Ophthalmologic Department for examination four days later because the disturbance of mental space representation became disabling: she got lost at home or in her neighbourhood, always going in a direction opposite to the one she was expected to go, because she reversed places. The diagnosis of RCS was confirmed, as the patient reversed the figures around a clock and drew the hands in the opposite direction of what was required according to the hour specified by the examiner. Concurrently, she had great difficulty reading the hour on a clock. Spatial organisation was also altered. Besides getting lost in her home or her neighbourhood, she reversed the major places on a map. A course of rehabilitation sessions was initiated to improve these difficulties. It consisted in different exercises. In the first period, the patient had to draw clock hands according to the instructions that were given to her. Later, when she was successful with this task, she had had to draw a map of known locations or to orientate herself on a grid to detect and to mark letters following arrows (Fig. 2). With the assistance of this rehabilitation, the clinical manifestations of RCS disappeared gradually in approximately 6 weeks. Her mental imagery disorder and deficit of inner space representation improved. Bu they are still noticeable 18 months later, especially when the patient is in crowded places or when she is tired.

## 2. Discussion

The RCS is usually the consequence of a lesion affecting the right cerebral hemisphere although Jones reported a patient with typical clinical manifestations of reversed clock phenomenon due to a left parietal lesion [1,2]. But, it does not seem to be related to a specific cortical or subcortical location. It can be due to a lesion that disrupts any components of the complex cerebral network with a right dominant network responsible for spatial attention and visuospatial tasks [3,4]. Kumral hypothesizes that the disruption will especially affect the systems of perception or motor exploration based on allocentric (or external world) coordinates [1]. Our patient presented a RCS and a space representation disorder due to a right internal parieto-occipital and hippocampal lesion. Such a vascular lesion disrupts the connections between the cerebral areas involved in the topographic analysis of external places and navigation. The precuneus, the right inferior parietal and bilateral medial parietal regions are included in the neural network of egocentric navigation [4–6]. The right hippocampus is important to locate places according to allocentric coordinates and to navigate accurately between them [6]. It is also involved in both the encoding and the retrieval of topographical memory and in the encoding of salient object location in association with the posterior cingulate gyrus, and bilateral occipital and occipito-temporal regions although left hippocampus seems to be involved in non-spatial aspect of navigation [3–7]. This RCS can be put together with mirror writing that usually affects the left hand and mirror reading. These phenomenons are observed in patients with left parieto-occipital lobe dysfunction. Mirror writing could be due to the activation of the left hemisphere spatial system that fails to translate the right motor programs to the left hand, although mirror reading could be a reversal of the left-to-right scanning process [8,9]. In our case, the patient did not present any reversal of the writing or reading patterns, and this absence could be explained by dissociation between representations of words based on the specific role of the right hemisphere and 2-dimensional visual objects based on the role of the left one.

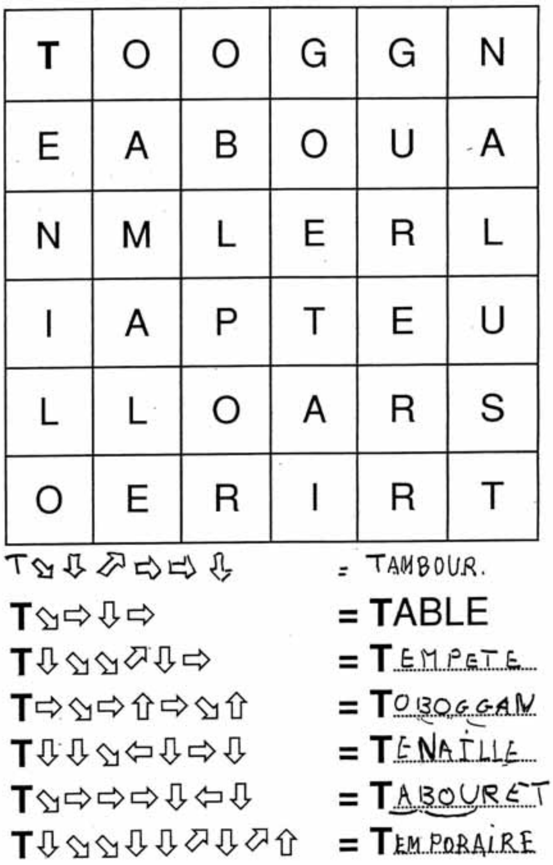

Fig. 2. n°2: One of the localization tasks. The patient had to find different letters of a word, starting with the letter "T" on the right-top box. The movement to reach the next letter is indicated by the arrows. Results at the end of the rehabilitation sessions.

In most cases, the clinical manifestations of the RCS disappear within a two weeks period [1]. However, the mechanisms underlying the restoration of neurological function after a stroke are still incompletely understood. Positron emission topography and functional MRI studies reveal the recruitment of cerebral territories not normally involved in the disrupted task to make up for the functional deficit [10, 11]. Additional mechanisms include enhancement of activity in preexisting networks and involvement of the ipsilesional network [12]. Thus, when stroke is limited to one or two functional territories, recovery can be fast since the compensation mechanisms involve only few structures. Such compensation should be difficult when many territories of the network are altered. We assume that our patient's recovery was longer than expected, due to the localization of her stroke. However, rehabilitation sessions may have been beneficial in the recovery process as they are in other cognitive deficits [13,14]. Various exercises were carried out. During a first period, the patient was asked to draw clocks according to instructions. Later, the exercises required orientation on a grid or a map using external information (arrows) or internal data (to set famous places on a map).

## 3. Conclusion

RCP is an uncommon complication of strokes often unrecognized as it usually improves in a few days. Psychovisual testing is valuable to display such a trouble when patients complain of undefined visual troubles. When this phenomenon persists, rehabilitation sessions based on localization exercises may be helpful.
